# Cerner Millennium’s Care Pathways for Specialty Care Referrals: Provider and Nurse Experiences, Perceptions, and Recommendations for Improvements

**DOI:** 10.1007/s11606-023-08285-2

**Published:** 2023-10-05

**Authors:** Kristina M. Cordasco, Alicia R. Gable, David A. Ganz, Julian W. Brunner, Anita J. Smith, Brian Hertz, Edward P. Post, Gemmae M. Fix

**Affiliations:** 1 VA HSR&D Center for the Study of Healthcare Innovation, Implementation and Policy, VA Greater Los Angeles Healthcare System, Los Angeles, CA USA; 2 Department of Medicine, VA Greater Los Angeles Healthcare System, Los Angeles, CA USA; 3 Department of Medicine, David Geffen School of Medicine at UCLA, Los Angeles, CA USA; 4 RAND Corporation, Santa Monica, CA USA; 5 VA Spokane Healthcare System, Spokane, WA USA; 6 Edward Hines Jr. VA Hospital, Hines, IL USA; 7 Loyola University Chicago Stritch School of Medicine, Maywood, IL USA; 8 VA Central Office, Washington, DC USA; 9 Ann Arbor VA Healthcare System, Ann Arbor, MI USA; 10 University of Michigan Medical School, Ann Arbor, MI USA; 11 Center for Healthcare Organization & Implementation Research, VA Bedford Healthcare System, Bedford, MA USA; 12 Department of Medicine, Boston University Chobanian & Avedisian School of Medicine, Boston, MA USA; 13 Department of Health Law Policy & Management, Boston University School of Public Health, Boston, MA USA

**Keywords:** referrals, electronic health records, user-centered design, qualitative evaluation, Veterans Administration

## Abstract

**Background:**

Using structured templates to guide providers in communicating key information in electronic referrals is an evidence-based practice for improving care quality. To facilitate referrals in Veterans Health Administration’s (VA) Cerner Millennium electronic health record, VA and Cerner have created “Care Pathways”—templated electronic forms, capturing needed information and prompting ordering of appropriate pre-referral tests.

**Objective:**

To inform their iterative improvement, we sought to elicit experiences, perceptions, and recommendations regarding Care Pathways from frontline clinicians and staff in the first VA site to deploy Cerner Millennium.

**Design:**

Qualitative interviews, conducted 12–20 months after Cerner Millennium deployment.

**Participants:**

We conducted interviews with primary care providers, primary care registered nurses, and specialty providers requesting and/or receiving referrals.

**Approach:**

We used rapid qualitative analysis. Two researchers independently summarized interview transcripts with bullet points; summaries were merged by consensus. Constant comparison was used to sort bullet points into themes. A matrix was used to view bullet points by theme and participant.

**Results:**

Some interviewees liked aspects of the Care Pathways, expressing appreciation of their premise and logic. However, interviewees commonly expressed frustration with their poor usability across multiple attributes. Care Pathways were reported as being inefficient; lacking simplicity, naturalness, consistency, and effective use of language; imposing an unacceptable cognitive load; and not employing forgiveness and feedback for errors. Specialists reported not receiving the information needed for referral triaging.

**Conclusions:**

Cerner Millennium’s Care Pathways, and their associated organizational policies and processes, need substantial revision across several usability attributes. Problems with design and technical limitations are compounding challenges in using standardized templates nationally, across VA sites having diverse organizational and contextual characteristics. VA is actively working to make improvements; however, significant additional investments are needed for Care Pathways to achieve their intended purpose of optimizing specialty care referrals for Veterans.

## BACKGROUND

Communication between providers during the specialty care referral process is a key aspect of providing clinically integrated care.^1^ Referrals require close, and ideally bi-directional, communication between the referring provider (e.g., primary care) and specialty care.^1–4^ Deficiencies in communication can result in inefficiencies, patient dissatisfaction, care delays, and adverse patient outcomes.^5–7^ In healthcare systems globally, electronic health record (EHR) tools have become integral to supporting these communications, resulting in more appropriate and efficient use of specialty care resources as well as improved communication, quality, safety, and patient satisfaction.^1,8–14^

The Veterans Health Administration (VA) is transitioning its EHR from Computerized Patient Record System (CPRS) to Cerner Millennium, with initial deployment having occurred in October 2020 in the Spokane VA Healthcare System (HCS). Although this transition is a potential opportunity to improve VA’s EHR-based referral communications, previous implementations of EHR-based referral tools have experienced significant barriers.^15^ Referrals are a complex process, vulnerable to disruptions in established processes, which are inherent in EHR transitions.^16^

In VA’s pre-transition state, using CPRS, referring providers convey to specialists the reasons for referral and, to facilitate referral triage, provide pertinent clinical information via templates, including specialists’ requirements and recommendations for pre-referral diagnostic and therapeutic management.^17^ This information exchange and clinical decision support, evidence-based practices for EHR-based referrals,^17,18^ are aimed at facilitating effective triage of referrals, as well as optimizing care efficiency through the patient having started initial therapeutics and/or having the results of studies by the first specialist visit. Each VA HCS has its own version of CPRS, with 139 instances nationally; templates are constructed locally, collaboratively designed by primary and specialty care providers in conjunction with negotiated local service agreements.

VA and Cerner have embarked upon an effort to create a similar mechanism for referral information exchange and clinical decision support for VA’s version of Cerner Millennium. “Care Pathways”—templated electronic forms, capturing needed information and prompting providers to order any missing pre-referral tests—have been created. Each specialty care service has one Care Pathway, with sub-paths for distinct conditions or needs. For example, there is one Care Pathway for cardiology referrals, with sub-paths for different cardiac problems (e.g., heart failure, arrhythmia). Sub-paths require referring providers to answer questions about patients’ medical conditions, mostly via checkboxes but also filling in dates; a text box is available at the end for additional information. Recommended pre-referral tests, if not already completed, are displayed as potential orders. To place a referral, providers must fill in the indicated Care Pathway; nurses can fill in these templates, proposing them for provider signature. Requestors must also route referrals to the appropriate VA facility, using station numbers. Cerner Millennium differs from CPRS in that it is a single instance across all VA sites and the Department of Defense. Therefore, Care Pathways are being standardized for national use; local site variations are not possible. Each Care Pathway is designed and iteratively improved by a workgroup, comprised of specialist providers from the respective specialty’s community of practice, in dialogue with facility staff.

The Healthcare Information Management and Systems Society (HIMSS) has published nine attributes for usable EHRs—they are simple, natural, consistent, and efficient; provide forgiveness and feedback; use language and present information effectively; preserve information context; and minimize cognitive load.^19^ To realize these attributes, EHR applications need to be refined through iterative assessments and improvements, consistent with health information technology (HIT) user-centered design principles.^20,21^ To inform ongoing iterative efforts to develop and improve upon Cerner Millennium’s referral processes, in the second year of its deployment, we elicited experiences, perceptions, and improvement recommendations for Care Pathways from Spokane HCS providers and nurses.

## METHODS

### Setting

The Spokane HCS consists of the Mann-Grandstaff VA Medical Center (VAMC)—located in Spokane, Washington—providing primary, specialty, and hospital care, as well as six affiliated community-based outpatient clinics—located across Washington, Idaho, and Montana—providing primary care services only. This arrangement is typical for VA HCSs. In VA, primary care is delivered via Patient Aligned Care Teams (PACTs), VA’s version of the patient-centered medical home model, in which primary care providers (PCPs) are closely supported by registered nurses (RNs) serving as care managers.^22^ VA uses five tiers to categorize “complexity” of its healthcare systems based on services available, patient population, and education and research activities;^23^ the Spokane HCS is in the lowest complexity tier.

### Design and Sample

We interviewed PCPs, PACT RNs, and specialty providers requesting and/or receiving referrals in the Spokane HCS. To interview providers experienced in placing referrals in Cerner Millennium, we queried VA’s Corporate Data Warehouse and invited all PCPs who had placed 30 or more referrals in the first 12 months of Cerner Millennium deployment. To identify nurses and specialists, VA Spokane HCS leaders provided us with names of, and we sent interview invitations to, all primary care nurses and cardiology, pulmonary, neurology, and general surgery specialists, which are core specialty services at the facility.

### Interview Guides

The interview guides, tailored for the interviewee’s role (PCP, specialist, nurse), were designed to explore adherence to published recommendations for optimizing EHR referrals,^18^ based on the socio-technical model for assessing HIT.^24^ We asked all interviewees about potential recommendations for improvements in Cerner Millennium tools and processes across the specialty care referral lifecycle (placing, tracking, and receiving referral results), overall impressions, and comparisons to CPRS. Specialists were asked about placing referrals to other specialists as well as, when receiving referrals, the quality of the information relayed to them for triage, and whether indicated pre-referral tests were ordered. All questions were followed by responsive probes to encourage interviewees to provide details. Specific to Care Pathways, participants were asked to provide an overview of the processes they used to place (or, for nurses, propose) referrals, impressions of how well Care Pathways work, and any alternative processes used to place referrals outside of Care Pathways.

### Data Collection

Interviews were initiated 1 year after Cerner Millennium’s initial deployment in the Spokane HCS, allowing time for interviewees to gain ample experience using a new technology and for deployment of significant initial software upgrades. Interviews were conducted and recorded via Microsoft Teams, without video, with PCPs in November 2021–January 2022 and specialty providers in January–February 2022. Nurse interviews, conducted in June and July 2022, were added in response to PCPs relaying that nurses played a significant role in placing and managing referrals. All identified providers and nurses were invited to participate. Recruitment was conducted via emails and Teams messages. Participation was voluntary and confidential; participants did not receive compensation. Interviews were professionally transcribed, except for one interview in which the participant declined recording and detailed notes were taken. All procedures received approval from the VA Greater Los Angeles and VA Puget Sound Institutional Review Boards.

### Analyses

To provide timely feedback to VA and Cerner leaders working to refine Care Pathways, we utilized rapid qualitative analysis.^25^ A structured template was developed from the interview guide, delineating topics covered by interview questions. Using this template, two investigators (KC, AG) independently summarized each transcript, using bullet points to capture the main points that were made within each topic. KC and AG then met to compare and merge their summaries via consensus into a final summary document for each transcript. Next, KC and AG collaboratively engaged in a constant comparison process of sorting bullet points on the topic of Care Pathways, across summaries, into themes (i.e., bullet points describing a similar issue) and, when appropriate, valence (positive, negative, neutral).^26^ These bullet points were then placed into a matrix, using Microsoft Excel, organized by themes and individual participants (de-identified).^27^ In a team-based analytic approach, health services researchers (KC, AG, DG, JB, GF), physicians with primary care experience (KC, DG, BH, EP), a Spokane-based nurse with primary care and patient safety experience (AS), and VA leaders who developed and are iteratively refining Care Pathways (BH, EP) reviewed this matrix to identify and discuss meta-themes.

## RESULTS

We invited 40 PCPs, 8 specialists, and 23 primary care nurses to participate. Of these, 19 (48%) PCPs, 4 (50%) specialty physicians, and 11 (48%) nurses responded and were interviewed. PCPs included 9 physicians and 10 nurse practitioners/physician assistants. All PCPs, 2 (50%) specialists, and 4 (36%) nurses had used CPRS; the remainder had started working in VA shortly before or after Cerner Millennium was launched. At the time of their interviews, participating PCPs, specialists, and nurses had used Cerner Millennium for an average of 13 (range 12–15), 15 (range 14–16), and 15 (range 5–20) months, respectively. Although not specifically asked, 11 (32%) participants spontaneously mentioned having used non-CPRS EHRs in past positions.

### Overall Impressions

Some interviewees liked aspects of the Care Pathways, expressing appreciation for their premise and logic. However, providers and specialists consistently reported frustration, describing the Care Pathways as time-consuming, cumbersome, non-intuitive, complicated, and not helpful for conveying information to specialists. Compared to PCPs and specialists, nurses generally reported having more favorable overall impressions, but offered similar critiques. Specialists reported not getting the information they needed for triaging, and that indicated tests were not ordered by referring providers. Interviewees commented on ways in which the Care Pathways, as currently configured, were problematic across multiple HIMSS usability attributes,^19^ described below.

### Efficiency

There was consensus that Care Pathways were a significant impediment to efficient workflow and diverted time from other patient care activities. Interviewees strongly recommended that Care Pathways be revised to decrease the time it takes to navigate through them. Although there was variation, respondents typically reported that it took “5 min” (ranging from “1–2 min” to “15 min”) to place a referral; some noted that the time burden was generally less for Pathways they used often and had become familiar with. This time burden was particularly problematic when patients needed multiple referrals:

If you have three or four referrals … that’s going to cause a bit of a slowdown in between patients and right now we’ve been given time for that but I just kind of wonder…how am I ever going to increase back to what I was able to do before Cerner? (PCP).

A significant contributor to the time burden was ascribed to Care Pathways having significantly more clicks, questions, and steps, compared to CPRS referral processes. Although a few nurses reported liking how checkboxes minimized typing, more commonly, interviewees reported that they impeded workflow, perceiving them as redundant and/or administrative (non-clinical). Providers reported needing to spend time searching through the EHR for the details being requested.

“I see a lot of checking the box that is not enhancing the care activity. It’s just making it easier for people who like to take data.” (PCP)

Interviewees recommended streamlining the Care Pathways by decreasing the number of clicks, questions, and steps; employing more auto-population for data available from the medical record; and, for pre-requisite testing guidelines, considering information boxes in place of checkboxes:

“A lot of the variations that they have I’ve already put in my consult write-up, so it ends up making it redundant. Ones that have fewer variations and just get right to the point—it’s much better. Others like to add information that you have to click off. For instance, they say: ‘Have you thought of this?’, ‘Did you order this study?’… Some of those are helpful. I don’t know that you should have to click off on them. They should probably just come up with an information window that you read and say, ah, have I thought of these things?” (PCP).

Another contributor to time burden, and a common source of frustration, was that, once in the Care Pathways, requestors were unable to access other portions of the EHR to look up the information being requested (e.g., lab values, dates) without exiting the Pathway. While this is also a limitation in CPRS, a commonly used workaround is to open a second CPRS session, which is not possible in Cerner Millennium. Instead, interviewees described a process by which they first accessed a Care Pathway to take notes on the details needed; then reviewed the EHR to obtain this information, recording it on a Microsoft Word document; and then re-entered and completed the Care Pathway.

Providers reported strategies for mitigating the time burden, including requesting their nurses to fill in the Care Pathways, which they then reviewed and signed; placing referrals between seeing patients (rather than with the patient present); using a “General Services” request or other mechanisms to circumvent using Care Pathways; and/or waiting until the end of the clinic session to place referrals. One interviewee described how delays in placing referrals, due to their time burden, presented a patient safety vulnerability:

“Some of these [Pathways] are so long-winded, and this gives the chance to drop the ball because you don’t get to do the paperwork at that time when you’re [seeing the patient].” (PCP)

### Simplicity, Naturalness, Effective Use of Language, and Consistency

Interviewees reported that too many Pathway options, non-intuitive organization, unfamiliar nomenclature, and a rudimentary search function made it difficult and time-consuming to find the appropriate pathway and/or sub-pathway. For some Pathways, the options available did not fit with local configurations and mix of specialty services available at the Spokane HCS. In some, the nomenclature being used for Pathway names was different from that previously used, with the search function not recognizing equivalent or related terms (e.g., “ENT” versus “Otolaryngology”). Referral needs were sometimes organized under services that were not intuitive to the referring providers, such as referrals for home health aides being under “Geriatrics and Extended Care” and home physical therapy referrals being under “Purchased Skilled Home Care.” Some interviewees described keeping a “cheat sheet” to remember which pathway to use for various needs.

“I liken it to exploring a cave. And then you get to your location, and you hope you remember next time how you got there, and it depends on how frequently you’re requesting that specific service …you have to pretty much know where you want to go before you start.” (PCP).

A few nurses found the embedded questions useful for conveying information, helping narrow down the specific purpose of the referral. However, more commonly, interviewees relayed that Pathway questions did not fit the situation or the information that needed to be conveyed; many questions asked for clinical information irrelevant to the patient’s condition or reason for referral, while other times relevant questions were missing.

“It’s not detailed when you need it to be, and it’s over detailed when you need it simplified.” (Specialist)

Interviewees also noted lack of fit between the tests being recommended by the Pathways and those expected by local specialty care providers, with inconsistencies across specialties. One specialist described frequently sending referrals back to the requesting provider asking for additional tests and then resubmission; PCPs and nurses reported that this occurred frequently for certain specialties. On the other hand, in some cases, specialists relayed that tests recommended in the Pathways were unnecessary. One interviewee explained how recommendations being determined on a national level may not fit with local service configurations and practice patterns:

“They wanted to make it pretty much so that it’s universal … The problem with that is [that] it’s not going to fit every facility …. They’ve tried setting that up, but even in my own service line different people do different things and people have different capabilities at their institutions.” (Specialist).

### Cognitive Load

Some interviewees described the prompts for pre-referral testing as useful, decreasing cognitive load by serving as a “cheat sheet” for indicated tests, decreasing delays, and adding value to the Veteran’s first appointment; some remarked that they liked having orders “pop up” automatically for their signature. However, multiple providers reported that these prompts are not useful and redundant and impede workflow since they usually had already ordered needed tests, especially for referrals made frequently.

Interviewees also noted difficulties with the requirement to choose a diagnosis or problem-based sub-pathway. Patients sometimes had multiple reasons for being referred (e.g., having both heart failure and an arrhythmia, which were separate sub-pathways on the Cardiology Pathway). In addition, having to choose from diagnosis-specific sub-pathways was problematic when the diagnosis had not yet been established (e.g., some mental health referrals). After answering the required questions, providers reported augmenting this information by using the text box at the end of the Pathway. Some providers reported that they needed to “get creative,” deleting and replacing auto-populated information after having chosen inaccurate answers to bypass it.

Increased cognitive load for specialists receiving referrals was also noted. Specialists reported not receiving most of the Pathway data entered by referring providers and were often unsure of the referral question. They commonly engaged in manual record review, reading providers’ progress notes to retrieve information they needed.

“I usually have to go the orders, right click on the [specialty] referral, see who placed it, when they placed it, go back into the document section, go look at notes around that period of time, go see what the PCP’s notes or urgent care or whatever nursing note there is or see what the patient is being evaluated for.” (Specialist).

To mitigate impact on increased cognitive load and workflow inefficiencies, specialists described relying heavily upon referral coordination nurses to gather information. However, specialists cited receiving unclear and incomplete information as a patient safety concern. One specialist relayed how there was a patient who arrived for a procedure and, due to lack of specificity in the information available, the patient needed to clarify details on which procedure was needed. Addressing this patient safety issue was a top recommendation.

### Forgiveness and Feedback

Interviewees noted aspects of the Pathways lacked “forgiveness” if the user made an error and did not provide feedback alerting them to that error, resulting in potential care delays in Veterans’ care. Interviewees noted that some Pathways have required templates but no instructions indicating that these templates are needed or how to access them; if the template is not filled in, the referral is returned rather than processed.

“For a mammography, you have to know to click open the template and fill it out. It’s not something that automatically populates and opens for you. If you don’t, then the referral sits, and it gets kicked back and you have to do it again … if you don’t know about them, or you don’t know to open them, you can easily skip them or miss them and then the referral comes back to you and it’s been sitting, waiting for you to fill out some information.” (PCP).

Separately, interviewees expressed frustrations with needing to route referrals to their appropriate VA locations, not only as an extra step, but also as an opportunity for error, with some referrals getting misrouted if the user made a mistake.

## DISCUSSION

The overall design of VA Cerner Millennium’s Care Pathways for supporting referral processes, with protocolized electronic standardized referral templates that include decision support, and both structured and free-text fields, is an evidence-based practice for improving referral communications.^17,18^ However, interviews with frontline providers and staff at the initial site to deploy VA Cerner Millennium demonstrate that the Pathways, and the organizational policies and processes linked to them, need substantial revision across several HIMSS-defined attributes^19^ to achieve their intended purpose.

User-centered design utilizes end-user feedback to inform iterations across the development process; our work is a salient illustration of how this iterative process is critical to EHR application development. As described by the National Institute of Standards and Technology, a key tenet of user-centered design is to “engage users early and often,” starting with pre-implementation assessments of user workflows (e.g., ethnographic studies and cognitive walkthrough interviews) and establishing performance objectives, followed by using human behavior principles and heuristics to design the application,^28^ including pre-deployment iterative user tests and adaptations, and then conducting post-deployment testing and refinement.^21^ Due to time pressures and competing organizational and provider demands within the context of the early COVID-19 pandemic, pre-deployment testing iterations were truncated. Our findings illustrate the adverse impacts of not employing the full user-centered design process prior to deployment. In an effort led by two of the authors (BH, EP) with consultative assistance from the lead author (KC), VA is now working to begin rectifying this issue using these findings to review the Care Pathway templates, including the formatting, readability, and volume of questions; this collaboration illustrates the value of VA’s embedded research infrastructure.^29^ It will be crucial for VA to continue such collaboration, further employing a user-centered design process by evaluating the impact of these revisions and, ideally, engage in a comprehensive multidisciplinary usability assessment of the Care Pathways, incorporating the expertise of human factors professionals,^21^ with the expectation that continued iterative refinements will be needed.

Consistent with the socio-technical model for HIT that guided our interviews,^24^ our findings illustrate the importance of aligning EHR applications with organizational structures, policies, procedures, and culture. Cerner Millennium presents a substantial shift in how VA users interact with the EHR for referrals; in advance of deployment, local VA sites need to consider how to adjust their staffing configurations, workflows, service agreements, and other organizational structures and processes, to align with Care Pathways. Further, it is telling that, although our interviews were conducted more than 1 year after deployment, providers and nurses were still adjusting to unfamiliar nomenclature and processes. Although pre-deployment considerations for structural, policy, and process adjustments, and bolstering of user training and support, may mitigate some of these issues, future VA deployment sites need to be prepared for this transition to have an extended impact on clinical productivity while the organization makes ongoing adjustments.

Our interviews also illustrate the need for Care Pathway enhancements within the technical domains of the socio-technical model for HIT.^24^ Namely, inability to exit a Care Pathway to navigate to other parts of the EHR significantly increased requestor time burden; fixing this technical feature was a top recommendation. In addition, interviewees reported manually inputting information into Pathways’ structured data fields, such as dates and test results, which is inefficient and increases opportunities for error. Using automation to better pre-populate these data is a key recommendation for optimizing referrals to improve safety and efficiency and decrease staff cognitive load.^17,18^ Separately, the premise of using templated EHR-based referrals is to achieve more effective information transfer; however, specialists reported not receiving most Care Pathway data. A recent enhancement, made after our interviews were conducted, renders Care Pathway information into the referring providers’ progress notes, making it now available to specialists. Assessing the effect of this advance, and potential need for additional technical improvements, should be a priority. Finally, the need to manually route a referral to the appropriate facility adds time, cognitive load, and opportunity for error. Interim steps have significantly automated routing based on decision logic specified by local facility leadership; nonetheless, addressing such technical limitations may require additional financial investments and will be paramount for optimizing patient safety and care efficiencies.

Cerner Millennium is a paradigm shift for standardizing processes across VA. In CPRS, local VA HCSs negotiate and construct referral templates; in Cerner Millennium, templates are universal across HCSs. Health care organizations having a history of allowing local sites flexibility in determining their own processes, as VA has, generally experience more difficulty with implementing standardized EHRs.^30^ However, this history of local flexibility arises from VA spanning 139 distinct HCSs across the nation, which varying organizational structures and mechanisms for providing care, reflecting the needs and resources available in their respective rural and urban communities. Therefore, while standardizing processes is generally associated with care improvements,^31^ there may also be adverse impacts.^32^ In some VA sites and specialties, the standardization that Cerner Millennium is prompting could result in a degradation of referral communications, compared to using locally tailored CPRS templates. Future studies should examine this issue carefully, both to guide VA decision-making, and also to contribute generalizable knowledge about the benefits and costs of standardization versus customization of EHR-based referrals processes within multi-site integrated health care systems that, like VA, have diverse organizational and community characteristics.

We noted that, while offering similar critiques of the Care Pathways, nurses’ impressions were overall more favorable compared to those of PCPs and specialists. Reasons for this difference may be multi-factorial, with nurses and providers having different roles, workflows, culture, and training. Nurse interviews also occurred approximately 6 months after those of providers. Further, almost two-thirds of the nurses had never used CPRS, having started working in VA after Cerner Millennium deployment. Future studies should assess for potential differences in Care Pathway perceptions based on user profession, discipline, role, experience, and other characteristics.

It is important to note the limitations of our work. The Spokane VA HCS, as a smaller and less complex VA HCS, has fewer trainees and in-house specialty care services than most; future assessments should include larger, more complex VA HCSs. Just under half of providers and nurses invited agreed to be interviewed; we do not know how the perspectives of those who agreed compared to those who did not. In addition, this evaluation occurred in VA’s Cerner Millennium’s initial deployment site; at the time of deployment, there had not been adequate time to fully develop training materials, some Pathways were still in development, and some had technical glitches. The extent to which these early frustrations continued to influence our interviewees’ impressions of the current functionality of the system is unknown.

In summary, although there is good evidence for the principles underlying VA Cerner Millennium’s Care Pathways, in their current deployment, user-centered design problems, lack of alignment with organizational characteristics, and technical limitations are interacting with challenges in standardizing practices across diverse VA sites. Significant additional investments are needed for Care Pathways to achieve their intended purpose of optimizing specialty referral processes for Veterans. In addition, VA needs to invest in preparing for, supporting, and assessing the impacts of the substantial associated shifts in referral processes and workflow that will be impacted by the transition to Cerner Millennium.

## Data Availability

The data generated during this study is not publicly available in order to preserve participant privacy, but may be made available upon reasonable request to the corresponding author.
